# Mucosal Infections and Invasive Potential of Nonencapsulated *Streptococcus pneumoniae* Are Enhanced by Oligopeptide Binding Proteins AliC and AliD

**DOI:** 10.1128/mBio.02097-17

**Published:** 2018-01-16

**Authors:** Jessica L. Bradshaw, Haley R. Pipkins, Lance E. Keller, James K. Pendarvis, Larry S. McDaniel

**Affiliations:** a Department of Microbiology and Immunology, University of Mississippi Medical Center, Jackson, Mississippi, USA; b School of Animal and Comparative Sciences, Bio5 Institute, University of Arizona, Tucson, Arizona, USA; UCLA School of Medicine

**Keywords:** NESp, nonencapsulated, *S. pneumoniae*, *Streptococcus pneumoniae*, colonization, otitis media, pneumococcal invasive disease, pneumococcus, virulence factors

## Abstract

Nonencapsulated *Streptococcus pneumoniae* (NESp) is an emerging human pathogen that colonizes the nasopharynx and is associated with noninvasive diseases such as otitis media (OM), conjunctivitis, and nonbacteremic pneumonia. Since capsule expression was previously thought to be necessary for establishment of invasive pneumococcal disease (IPD), serotype-specific polysaccharide capsules are targeted by currently licensed pneumococcal vaccines. Yet, NESp expressing oligopeptide binding proteins AliC and AliD have been isolated during IPD. Thus, we hypothesize AliC and AliD are major NESp virulence determinants that facilitate persistence and development of IPD. Our study reveals that NESp expressing AliC and AliD have intensified virulence compared to isogenic mutants. Specifically, we demonstrate AliC and AliD enhance murine nasopharyngeal colonization and pulmonary infection and are required for OM in a chinchilla model. Furthermore, AliC and AliD increase pneumococcal survival in chinchilla whole blood and aid in resistance to killing by human leukocytes. Comparative proteome analysis revealed significant alterations in protein levels when AliC and AliD were absent. Virulence-associated proteins, including a pneumococcal surface protein C variant (CbpAC), were significantly downregulated, while starvation response indicators were upregulated in the double mutant relative to wild-type levels. We also reveal that differentially expressed CbpAC was essential for NESp adherence to epithelial cells, virulence during OM, reduction of C3b deposition on the NESp surface, and binding to nonspecific IgA. Altogether, the rise in NESp prevalence urges the need to understand how NESp establishes disease and persists in a host. This study highlights the roles of AliC, AliD, and CbpAC in the pathogenesis of NESp.

## IMPORTANCE

Despite the effective, widespread use of licensed pneumococcal vaccines over many decades, pneumococcal infections remain a worldwide burden resulting in high morbidity and mortality. NESp subpopulations are rapidly rising in the wake of capsule-targeted vaccine strategies, yet there is very little knowledge on NESp pathogenic potential and virulence mechanisms. Although NESp lacks a protective capsule, NESp lineages expressing AliC and AliD have been associated with systemic infections. Furthermore, higher antibiotic resistance rates and transformation efficiencies associated with emerging NESp threaten treatment strategies needed to control pneumococcal infections and transmission. Elucidating how NESp survives within a host and establishes disease is necessary for development of broadened pneumococcal prevention methods. Our study identifies virulence determinants and host survival mechanisms employed by NESp with a high pathogenic potential. Moreover, our study also identifies virulence determinants shared by NESp and encapsulated strains that may serve as broad prevention and therapeutic targets.

## INTRODUCTION

*Streptococcus pneumoniae* (pneumococcus) colonizes the human nasopharynx and causes a wide variety of diseases such as otitis media (OM), pneumonia, bacteremia, and meningitis following dissemination from the nasopharynx (1, 2). Notably, pneumococci are the leading cause of bacterial pneumonia, OM, and bacterial meningitis and are the primary reason for unscheduled pediatrician visits for children less than 5 years old (3, 4). Additionally, high morbidity and mortality are associated with pneumococcal infections in the elderly (5). Preventative measures against pneumococcal disease include a 13-valent pneumococcal conjugate vaccine (PCV) and 23-valent polysaccharide vaccine that target specific polysaccharide capsules of the pneumococcus (6). Since there are 97 known pneumococcal serotypes, the current vaccines confer protection against a limited number of serotypes (7). Furthermore, pneumococcal vaccines do not prevent pneumococcal disease caused by nonencapsulated *S. pneumoniae* (NESp), as this subpopulation lacks capsule expression. Although substantial decreases in disease associated with serotypes included in the current vaccines have been observed, an increase in the isolation of nonvaccine serotypes and NESp have been reported following the use of these vaccines (8–11). Consequently, selective pressure against the polysaccharide capsule provided by current vaccines has likely driven the increase in NESp colonization and disease establishment.

Three genes (*pspK*, *aliC*, and *aliD*) that replace capsule synthesis genes encoded in the capsule polysaccharide biosynthetic (*cps*) locus have been identified in NESp strains (12, 13). The replacement of capsule genes with novel genes in NESp suggests that the proteins encoded by these genes provide functions that may compensate for lack of capsule production. PspK has been described as an adhesin that allows NESp to colonize as efficiently as encapsulated pneumococci (14, 15). Remarkably, extensive surveillance of invasive pneumococcal disease (IPD) isolates has revealed an association of invasive disease with NESp expressing oligopeptide binding proteins AliC and AliD despite the absence of a protective, antiphagocytic capsule thought to be necessary to establish IPD (12, 16–20). A survey of pneumococcal isolates in Switzerland from 1998 to 2002 first exposed the isolation of NESp expressing AliC and AliD from a blood sample (12). Surveillance of IPD isolates in the United States from 2006 to 2009 identified that 67% of pneumococcal isolates lacking *cps* genes encoded both *aliC* and *aliD* in their *cps* locus (19). In a surveillance study spanning 2003 to 2013 in South Africa, 82% of NESp IPD isolates with complete deletion of *cps* genes were described as containing *aliC* and *aliD* in place of their *cps* genes (20). Collectively, the isolation of IPD-associated NESp expressing AliC and AliD from geographically distinct areas of isolation suggests that these oligopeptide binding proteins afford a selective advantage during invasive disease.

Sequence analyses and previous studies have indicated that AliC and AliD are substrate binding proteins that concentrate small, extracellular oligopeptides at the bacterial surface and deliver the substrates to transmembrane permeases for substrate import (12, 13, 21, 22). Analysis has also revealed AliC and AliD to be orthologous to outer membrane proteins of the OppA family found in Gram-negative bacteria (23, 24). Currently, a comprehensive understanding of how AliC and AliD influence NESp physiology and virulence has yet to be established. Oligopeptides are commonly imported by Gram-positive bacteria as sensory molecules that alter expression of numerous virulence factors, such as hemolysins, adhesins, and genes responsible for biofilm formation (25). Outer membrane proteins in Gram-negative bacteria that are homologous to AliC and AliD have been shown to be significant effectors of nutrient acquisition and complement evasion (24, 26). On the basis of IPD surveillance data and sequence homology analysis, we hypothesize that oligopeptide transport involving AliC and AliD enhances the pathogenic potential of NESp by altering protein expression that aids in bacterial persistence.

Here, we describe the impact of AliC and AliD on NESp virulence by examining *in vitro* virulence-associated phenotypes and investigating these oligopeptide binding proteins during *in vivo* models of pathogenesis. We demonstrate that AliC and AliD are significant factors in colonization of the murine nasopharynx and lungs, disease progression during OM in a chinchilla model, survival within chinchilla whole blood, and evasion of host immune responses encountered during invasive disease. We also reveal that a NESp surface adhesin CbpAC, which is homologous to encapsulated pneumococcal surface protein C, enhances virulence during mucosal infections and provides a host evasion mechanism.

## RESULTS

### NESp biofilm biomass is reduced by the presence of AliC and AliD.

**FIG 1:**
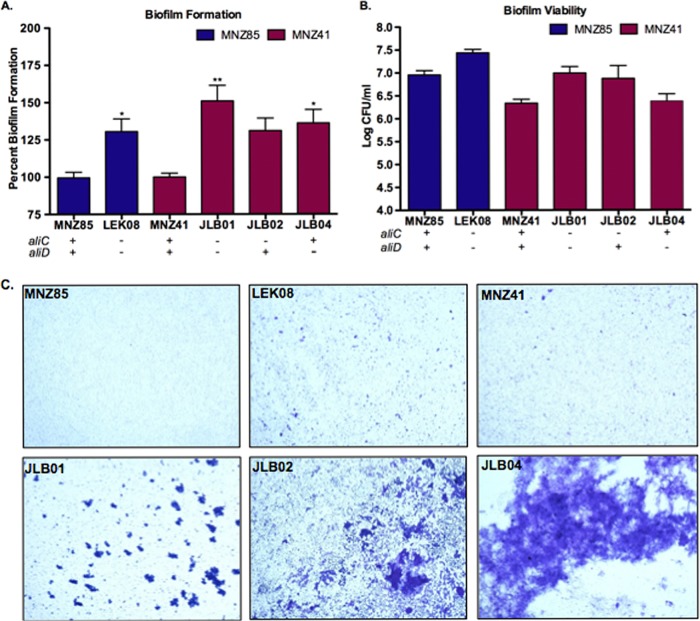
Biofilm biomass is reduced *in vitro* by the presence of AliC and AliD. (A) Biofilm formation was quantified at 24 h after seeding a 24-well plate with 10^5^ pneumococci and corresponds to wild-type strains MNZ85 and MNZ41 set to 100% formation. The strain backgrounds are indicated by the color key. (B) Pneumococcal viability in the biofilm was assessed at 24 h by collecting biofilms and plating on BA to enumerate CFU. In panels A and B, values are means plus standard errors of the means (error bars) from at least two independent studies performed in triplicate. Values that are significantly different are indicated by asterisks as follows: *, *P* < 0.05; **, *P* < 0.01. (C) Micrographs of 24-h biofilms stained with crystal violet and captured at ×10 magnification. Deletion of *aliC* and *aliD* significantly increased biofilm formation in both strain backgrounds (A and C) but did not affect NESp viability in biofilms (B).

Biofilm formation and pneumococcal viability were quantified to determine whether the absence of AliC or AliD resulted in differences in community growth phenotypes (Fig. 1; also see Fig. S1A in the supplemental material). We investigated NESp carriage isolates MNZ85 and MNZ41 of differing sequence types, 2315 and 6153, respectively (Table 1). Also, isogenic *aliC* and *aliD* double deletion mutants of strains MNZ85 and MNZ41, LEK08 and JLB01, respectively, were analyzed. Significant increases (*P* < 0.05) in biofilm formation (Fig. 1A) were observed when *aliC* and *aliD* were deleted in MNZ85 and MNZ41 backgrounds. Although biofilm formation increased with a single deletion of *aliC* or *aliD* in strain MNZ41 (Fig. 1A), only a single deletion of *aliD* significantly increased (*P* < 0.05) biofilm formation. Increased biofilm formation was not a direct result of increased pneumococcal viability in the biofilms, as there were no significant differences in NESp cell densities in either strain background when *aliC* and *aliD* were deleted (Fig. 1B). NESp aggregation and presence of biomass towers as visualized in stained biofilms were enhanced when *aliC* and *aliD* were absent from both backgrounds (Fig. 1C).

10.1128/mBio.02097-17.1 FIG S1  Genetic complementation restores *in vitro* phenotypes comparable to NESp wt strain MNZ41. MNZ41 isogenic mutant encoding *aliC* but not *aliD* was transformed with pABG5::*aliD* to produce JLB07 for complementation studies. (A and B) Biofilm formation (A) and adherence to human epithelial cells (B) were significantly decreased in strain JLB07 than those in the double mutant strain (JLB01) and similar to wt levels. (C and D) Hemolytic activity increased significantly in strain JLB07 compared to strain JLB01 and was similar to the wt (D), with a strong trend toward increased Ply production also observed (C). Data represent two independent studies performed in triplicate. Error bars represent standard errors of the means. Values that are significantly different are indicated by bars and asterisks as follows: *, *P* < 0.05; **, *P* < 0.01; ***, *P* < 0.001. Download FIG S1, PDF file, 0.2 MB. Copyright © 2018 Bradshaw et al. 2018 Bradshaw et al. This content is distributed under the terms of the Creative Commons Attribution 4.0 International license.

**TABLE 1:** Descriptions of pneumococcal strains used in this study

Serotype	Sequence type	Strain	Strain description or relevant genotype	Antibiotic resistance^a^	Reference
NESp	2315	MNZ85	Carriage isolate	ND	13
		LEK08	MNZ85 Δ*aliC* Δ*aliD*	Spec (300 µg/ml)	This study

NESp	6153	MNZ41	Carriage isolate	Tmp (50 µg/ml)	13
		JLB01	MNZ41 Δ*aliC* Δ*aliD*	Spec (300 µg/ml)	This study
		JLB02	MNZ41 Δ*aliC*	Kan (500 µg/ml)	This study
		JLB04	MNZ41 Δ*aliD*	Spec (300 µg/ml)	This study
		JLB10	MNZ41 Δ*cbpAC*	Spec (300 µg/ml)	This study

2	595	D39	Avery’s virulent isolate	ND	57
		ΔPAC	D39 Δ*ply* Δ*pspA* Δ*pspC*	Tmp, Tet, and Erm (10 µg/ml, 5 µg/ml, and 0.3 µg/ml, respectively)	31

19F	43	EF3030	Otitis media isolate	ND	58

a ND, not determined; Spec, spectinomycin; Tmp, trimethoprim; Kan, kanamycin; Tet, tetracycline; Erm, erythromycin.

### AliC and AliD diminish adherence to epithelial cells but do not affect NESp invasion rates.

**FIG 2:**
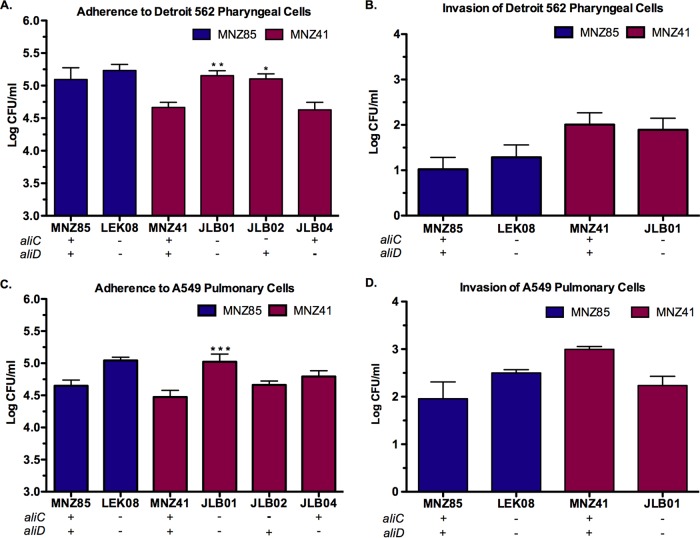
NESp adherence and invasion of human epithelial cells. Detroit 562 pharyngeal (A and B) or A549 pulmonary (C and D) epithelial cells were incubated with 10^7^ pneumococci. Adhered or intracellular bacteria were enumerated by plating on BA. A deletion of *aliC* and *aliD* in MNZ41 significantly increased adherence to both cell lines (A and C) but did not affect invasion into epithelial cells (B and D). Values are means plus standard errors of the means (error bars) from three independent studies performed in triplicate. Values that are significantly different are indicated by asterisks as follows: *, *P* < 0.05; **, *P* < 0.01; ***, *P* < 0.001.

Pneumococcal adherence and invasion of epithelial cells were examined to assess whether AliC and AliD altered bacterial interactions with host cells at the cell-to-cell interface (Fig. 2 and Fig. S1B). Two different human cell lines, Detroit 562 pharyngeal cells (Fig. 2A and B) and A549 pulmonary cells (Fig. 2C and D), were utilized to mimic two specific niches for colonization and establishment of pneumococcal disease. A deletion of *aliC* and *aliD* did not alter NESp MNZ85 adherence to pharyngeal cells (Fig. 2A) or pulmonary epithelial cells (Fig. 2B). However, MNZ41 adherence to pharyngeal and pulmonary cells significantly increased (*P* < 0.01) when *aliC* and *aliD* were deleted (Fig. 2A and C), with increased adherence to pharyngeal cells also being observed when *aliC* alone was deleted (Fig. 2A). MNZ85 and MNZ41 cells invaded pulmonary cells more efficiently than pharyngeal cells, but the presence of AliC and AliD did not alter invasion rates in either epithelial cell line (Fig. 2B and D).

### The intracellular level and cytolytic activity of pneumolysin are increased by the presence of AliD in virulent strain MNZ41.

**FIG 3:**
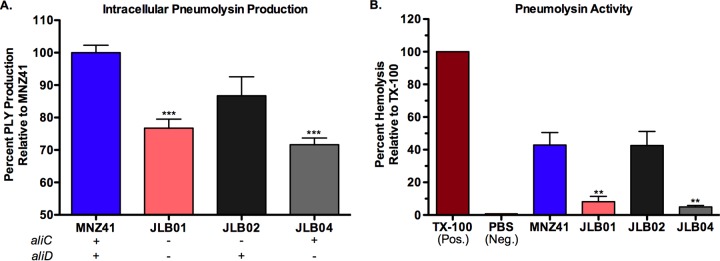
AliD impacts pneumolysin (Ply) levels and activity. (A) Intracellular Ply levels were determined by using equivalent protein concentrations of NESp lysates in an indirect ELISA, and Ply levels were standardized to the levels for strain MNZ41 set at 100%. Ply activity was quantified by incubating sheep red blood cells (RBCs) with standardized NESp lysates and measuring RBC lysis. Lysis by Triton X-100 (TX-100) was set at 100%, and PBS served as the negative control for lysis. In panels A and B, a deletion of *aliD* resulted in a significant decrease in Ply production and activity. At least two independent studies were performed, and each strain was tested in triplicate. Error bars represent standard errors of the means. Values that are significantly different are indicated by asterisks as follows: **, *P* < 0.01; ***, *P* < 0.001.

Pneumolysin (Ply) is a pneumococcal virulence factor that lacks a secretion signal and functions as a cholesterol-dependent cytolysin of host cells (27). Intracellular Ply levels were quantified by an enzyme-linked immunosorbent assay (ELISA) to determine whether oligopeptide transport through AliC and AliD affected Ply production (Fig. 3A and Fig. S1C). A deletion of both *aliC* and *aliD* resulted in a highly significant decrease (*P* < 0.001) in the intracellular Ply level compared to that of strain MNZ41, and this effect was also observed with a single deletion of *aliD* (Fig. 3A). A hemolysis assay was also utilized to determine whether differences in Ply levels correlated with cytolytic activity (Fig. 3B and Fig. S1D). Similar to ELISA results (Fig. 3A), lysing of red blood cells was significantly reduced (*P* < 0.01) when AliD was absent (Fig. 3B).

### AliC and AliD enhance murine nasopharyngeal colonization and pulmonary infection.

**FIG 4:**
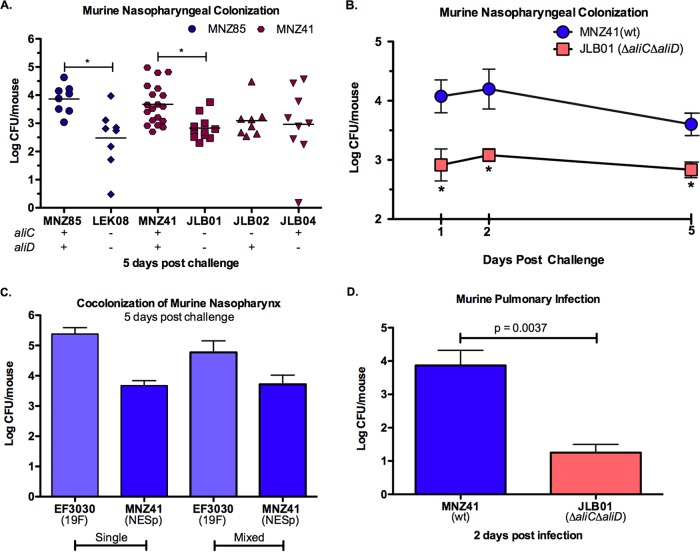
AliC and AliD enhance nasopharyngeal colonization and pulmonary infection of C57BL/6 mice. (A and B) Nasopharyngeal wash and tissue homogenates were plated on BA, and bacterial counts were combined to estimate log CFU/mouse at 1, 2, and 5 days after intranasal challenge with 10^7^ pneumococci. In panel A, each symbol represents the value for an individual mouse, and a short line represents the mean value for that group of mice. Mean values that are significantly different (*P* < 0.05) are indicated by a bar and asterisk. wt, wild type. (C) Mice were intranasally inoculated with encapsulated serotype 19F strain EF3030 and NESp strain MNZ41 at a 1:1 mixture of 10^7^ CFU or were inoculated with 10^7^ CFU of each strain independently. Replica plating on selective BA was used to enumerate recovered log CFU/mouse at 5 days postchallenge. (D) The lungs were collected 2 days after intratracheal infection with 10^7^ pneumococci, and lung homogenates were plated on BA to enumerate CFU. AliC and AliD significantly enhanced colonization of the murine nasopharynx (A and B) and infection of the lungs (D). NESp was able to efficiently colonize the murine nasopharynx despite cocolonization with encapsulated 19F strain (C). Each independent study was performed twice. Error bars represent standard errors of the means.

We utilized a C57BL/6 murine model to assess whether differences in adhesion correlated with the ability to colonize the murine nasopharynx and establish an active lung infection. Significantly higher counts of bacteria were recovered from the mouse nasopharynx when AliC and AliD were both present in the MNZ85 (*P* < 0.05) and MNZ41 (*P* < 0.05) backgrounds 5 days after intranasal challenge (Fig. 4A and Fig. S2A). A trend in decreased recovered bacteria was also observed when only *aliC* or *aliD* was deleted in strain MNZ41. To determine the impact of oligopeptide transport through AliC and AliD during earlier stages of colonization, we also assessed pneumococcal colonization 1 and 2 days after challenge (Fig. 4B). Bacterial loads in the nasopharynx were significantly higher (*P* < 0.05) 1 and 2 days after intranasal challenge when AliC and AliD were present, with a log unit difference in recovered bacteria observed as early as 1 day postchallenge (Fig. 4B). Since NESp was able to efficiently colonize the nasopharynx, we then tested the ability of NESp MNZ41 to colonize the nasopharynx when in competition with a prevalent encapsulated strain, EF3030 (serotype 19F). Five days after intranasal challenge, NESp CFU recovered from mice inoculated with a mixture of strains MNZ41 and EF3030 were similar to NESp CFU recovered from a single inoculation of MNZ41 (Fig. 4C). Although previous studies have established the ability of NESp to efficiently colonize the nasopharynx (12–14, 22, 28), there is very limited evidence as to whether NESp can persist in the lung environment. Two days after intratracheal inoculation of NESp, strain MNZ41 was recovered from the lungs of mice, and a deletion of *aliC* and *aliD* significantly reduced (*P* = 0.0037) bacterial load during an active lung infection (Fig. 4D).

10.1128/mBio.02097-17.2 FIG S2  *In vivo* murine nasopharyngeal colonization and chinchilla otitis media phenotypes are restored after genetic complementation. (A and B) Recovered JLB07 CFU during murine colonization at 5 days postchallenge (A) and chinchilla otitis media at 4 days postinfection (B) were significantly increased from those of mutant strain JLB01 and similar to wt levels. Data represent results of at least two independent studies. Error bars represent standard errors of the means. Values that are significantly different are indicated by bars and asterisks as follows: *, *P* < 0.05; ***, *P* < 0.001. Download FIG S2, PDF file, 0.1 MB. Copyright © 2018 Bradshaw et al. 2018 Bradshaw et al. This content is distributed under the terms of the Creative Commons Attribution 4.0 International license.

### Chinchilla acute OM is nearly abrogated when AliC and AliD are absent.

**FIG 5:**
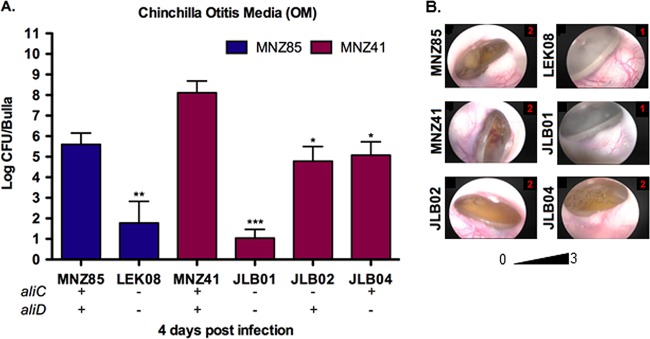
AliC and AliD increase NESp virulence during acute otitis media (OM) in chinchillas. (A) Chinchillas received intrabullar injection of 10^7^ pneumococci in each bulla. Middle ear effusion, bulla wash, and bulla homogenate of individual bulla were plated on BA, and bacterial counts were combined to estimate log CFU/bulla recovered at 4 days postinfection. (B) Representative otoscopic pictures of tympanic membranes of infected chinchillas at 4 days postinfection with the pathology score shown in red in the top rightmost corner of the picture. (A and B) Bacterial recovery and tympanic pathology were significantly reduced when *aliC* and *aliD* were deleted. The scoring rubric and combined tympanic pathology scores are given in Materials and Methods and in Table 2. At least four chinchillas (*n* = 8) were infected for each strain, and the data shown are representative of two independent experiments. Error bars represent standard errors of the means. Values that are significantly different are indicated by asterisks as follows: *, *P* < 0.05; **, *P* < 0.01; ***, *P* < 0.001.

Virulence during otitis media (OM) was assessed through otoscopic examination and biofilm formation scoring of the middle ear postmortem (Table 2 and Fig. 5B), as well as pneumococci recovered from each inoculated bulla 4 days postinfection (Fig. 5A and Fig. S2B). In a chinchilla model, OM was significantly attenuated when AliC and AliD were absent in both MNZ85 (*P* < 0.01) and MNZ41 (*P* < 0.001) backgrounds. Moreover, a single deletion of *aliC* or *aliD* resulted in significantly decreased recovery of bacteria (*P* < 0.05), but did not attenuate virulence as strongly as a deletion of both *aliC* and *aliD* (Fig. 5A). Increased tympanic pathology and biofilm formation were observed in wild-type (wt) strains compared to isogenic double deletion mutants, but a single deletion of *aliC* or *aliD* did not result in significantly different otoscopic or *in vivo* biofilm scores (Table 2).

**TABLE 2:** Chinchilla otitis media (OM) tympanic pathology and intrabullar biofilm scores

Strain	Otoscopic score^a^ (mean ± SEM)	Biofilm score^b^ (mean ± SEM)
MNZ85	1.64 ± 0.13	1.71 ± 0.13
LEK08	0.53 ± 0.19	0
MNZ41	2	2 ± 0.19
JLB01	0.5 ± 0.19	0.42 ± 0.19
JLB02	2	1.67 ± 0.28
JLB04	1.79 ± 0.11	1.71 ± 0.27
JLB10	1.75 ± 0.25	1 ± 0.57

a Otoscopic examination of the tympanic membrane was scored as follows: 0 for no visible inflammation, 1 for inflammation, 2 for effusion, and 3 for tympanic membrane rupture.

b Biofilm formation was scored as follows: 0 for no visible biofilm formation, 1 for biofilm formation on the surface of bulla, 2 for the biofilm traverses the bulla, and 3 for the biofilm traverses the bulla with thickening.

### Pneumococcal survival after exposure to chinchilla whole blood and human PMNs is mediated by AliC and AliD.

**FIG 6:**
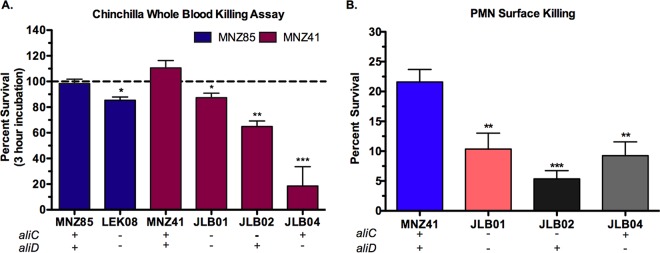
AliC and AliD promote survival during exposure to chinchilla whole blood and human polymorphonuclear (PMN) cells. (A) Percent survival in chinchilla whole blood was calculated by dividing the blood mixture bacterial counts at 3 h by the NESp inoculum and multiplying by 100. (B) Pneumococcal survival after 24-h exposure to human PMNs at a PMN:bacterium ratio of 800:1 was calculated by dividing the number of CFU of PMN-treated NESp by the number of mock-treated NESp and multiplying by 100. NESp CFU were enumerated by plating on BA. AliC and AliD significantly increased NESp survival during exposure to chinchilla whole blood (A) and human neutrophils (B). Data represent two independent studies performed in triplicate. Error bars represent standard errors of the means. Values that are significantly different are indicated by asterisks as follows: *, *P* < 0.05; **, *P* < 0.01; ***, *P* < 0.001.

During chinchilla OM, chinchillas infected with wt strains displayed signs of systemic disease (lethargy, ataxia, and unresponsiveness to sound), and NESp was recovered from the blood. To determine whether NESp was able to survive within uninfected chinchilla whole blood, bacterial strains were incubated in heparinized chinchilla whole blood. Percent bacterial survival was determined following a 3-h incubation (Fig. 6A). Deletion of *aliC* and *aliD* from either strain MNZ85 or strain MNZ41 resulted in decreased pneumococcal survival in chinchilla whole blood (*P* < 0.05), and MNZ41 bacterial counts increased during the incubation. A single deletion of *aliC* diminished bacterial survival (*P* < 0.01) compared to wt or double deletions. Additionally, a single deletion of *aliD* further diminished bacterial survival (*P* < 0.001), with almost all NESp cleared after 3 h of incubation in chinchilla whole blood. To investigate how NESp survives in whole blood, we examined pneumococcal survival when exposed to human neutrophils (polymorphonuclear leukocytes [PMNs]), the most abundant immune cell in blood. After 24 h of allowing PMNs and bacteria to interact on sheep blood agar (BA), pneumococcal survival was greatly reduced when *aliC* (*P* < 0.001) or *aliD* (*P* < 0.01) was deleted in the MNZ41 background (Fig. 6B).

### Oligopeptide transport involving AliC and AliD alters the physiological state of NESp and upregulates virulence-associated proteins.

*In vitro* and *in vivo* models of virulence revealed that a deletion of *aliC* and *aliD* altered phenotypes and attenuated NESp virulence. However, these phenotypic differences were not a result of differences in growth kinetics (Fig. S3). To examine how oligopeptide transport involving AliC and AliD impacts bacterial physiology and interactions with the host, we employed mass spectrometry to compare the proteomes of strains MNZ41 and JLB01. Proteomic analysis of pneumococcal strains MNZ41 and JLB01 yielded 186 and 112 proteins, respectively. The false-discovery rates ranged from 0.2% to 1.2% across all samples. Differential expression analysis (Data Set S1) showed that 75 proteins were significantly differentially expressed (*P* ≤ 0.05) between strains MNZ41 and JLB01. Specifically, 12 proteins were upregulated and 63 proteins were downregulated in strain JLB01 compared to strain MNZ41 levels. Proteins were grouped based on direction of regulation and function, and a representative list of differentially expressed proteins that may impact NESp persistence and virulence is listed in Table 3.

10.1128/mBio.02097-17.3 FIG S3  Growth curves of strains MNZ41 and JLB01 cultured in THY broth. Pneumococci were grown to mid-log phase and diluted to 100 CFU/ml in 10 ml THY for growth curve analysis. At indicated time points, 60 μl of culture was removed, diluted, and plated in duplicate to enumerate CFU/ml. There was no significant difference in growth between strains MNZ41 and JLB01. Data represent three independent studies. Errors bars represent standard deviations of the means. Download FIG S3, PDF file, 0.1 MB. Copyright © 2018 Bradshaw et al. 2018 Bradshaw et al. This content is distributed under the terms of the Creative Commons Attribution 4.0 International license.

10.1128/mBio.02097-17.4 DATA SET S1  Microsoft Excel file containing comparative proteome data and analysis. Download DATA SET S1, XLSX file, 0.1 MB. Copyright © 2018 Bradshaw et al. 2018 Bradshaw et al. This content is distributed under the terms of the Creative Commons Attribution 4.0 International license.

**TABLE 3:** Representative proteins dysregulated when AliC and AliD are absent from strain MNZ41

NCBI accession no.	Protein description	Protein function	Regulation direction in strain JLB01	*P* value
EPD18890.1	Adenylate kinase	ADP synthesis using ATP and AMP	Up	0.029
EPD19581.1	DNA protection during starvation protein	Oxidative damage protectant	Up	0.014
EDP19576.1	Endoribonuclease L-PSP	Translation initiation inhibitor	Up	0.014
EPD21090.1	Acetaldehyde reductase	Regeneration of NAD^+^	Up	0.014
EPD18727.1	Glutamyl aminopeptidase	Glutamate synthesis	Down	*P* < 0.01
EPD20852.1	Asparagine synthetase AsnA	Asparagine synthesis	Down	*P* < 0.01
EPD19911.1	Aspartate kinase	Threonine synthesis from aspartate	Down	0.012
EPD22312.1	IMP dehydrogenase	*De novo* synthesis of guanine nucleotides	Down	0.013
EPD21793.1	Uracil ribosyltransferase	Pyrimidine (uracil) salvage	Down	0.029
EPD18378.1	6-Phosphofructokinase	Synthesis of fructose-1,6-bisphosphate in glycolysis	Down	*P* < 0.01
EPD21778.1	Pyruvate oxidase (SpxB)	Production of endogenous H_2_O_2_, pneumolysin release	Down	0.014
EPD16767.1	GroEL	Refolding of misfolded proteins under stressful conditions	Down	*P* < 0.01
EPD19400.1	DnaK	Chaperone; folding of nascent proteins and misfolded proteins during stress	Down	0.014
EPD21629.1	NADH oxidase	Protects against oxidative stress, aids in adherence	Down	0.011
EPD17685.1	CbpAC (PspC variant)	Surface adhesion; binding to F_c_ IgA	Down	*P* < 0.01

### NESp PspC variant CbpAC increases adherence to epithelial cells and virulence during chinchilla OM.

**FIG 7:**
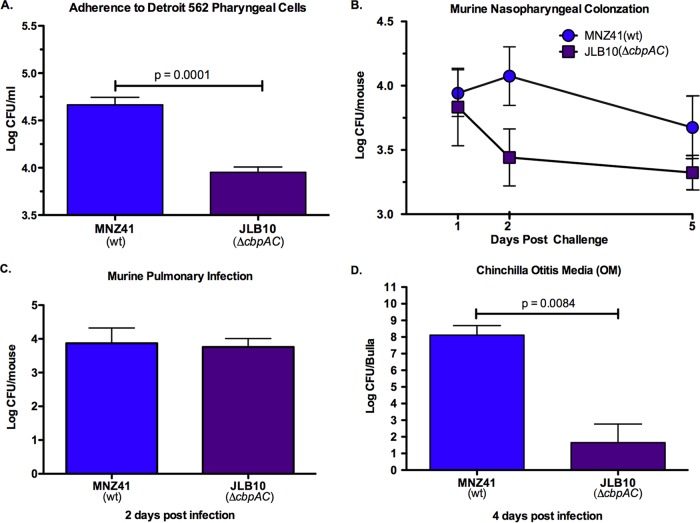
NESp CbpAC improves adherence to human epithelial cells and virulence during chinchilla OM. (A) Human Detroit 562 pharyngeal epithelial cells were incubated with 10^7^ NESp, and adhered pneumococci were enumerated by plating on BA. Adherence assays were performed in triplicate, and data are representative of two independent studies. (B) Nasal washes and tissue homogenates of C57BL/6 mice (*n* ≥ 7) intransally challenged with 10^7^ NESp were plated on BA, and bacterial counts were combined to estimate log CFU/mouse at 1, 2, and 5 days postinfection. (C) C57BL/6 mice (*n* ≥ 6) were intratracheally infected with 10^7^ NESp, and the lungs were collected at 2 days postinfection for bacterial enumeration. (D) Each bulla of chinchillas (*n* ≥ 4) was injected with 10^7^ NESp, and individual bulla effusion fluid, wash fluid, and homogenate were plated on BA to enumerate bacterial CFU at 4 days postinfection. Effusion, bulla, and wash bacterial counts were pooled for each bulla to estimate log CFU/bulla. CbpAC significantly increased adherence to pharyngeal epithelial cells (A) but was not required for colonization of murine nasopharynx (B) or infection of the lungs (C). (D) Deletion of *cbpAC* significantly attenuated OM in chinchillas. Error bars denote standard errors of the means.

Comparative proteomic analysis revealed that a variant of the encapsulated virulence protein PspC, described as CbpAC (29), was upregulated when AliC and AliD were present. We investigated whether CbpAC was essential for increased NESp virulence in the MNZ41 background. CbpAC significantly increased (*P* = 0.0001) adherence to pharyngeal cells (Fig. 7A) and was a significant virulence factor (*P* = 0.0084) during chinchilla OM (Fig. 7D). However, the NESp PspC variant did not significantly impact colonization of the murine nasopharynx (Fig. 7B) or infection of the murine lung (Fig. 7C).

### CbpAC prevents deposition of human complement component C3b and binds to nonspecific mouse IgA.

**FIG 8:**
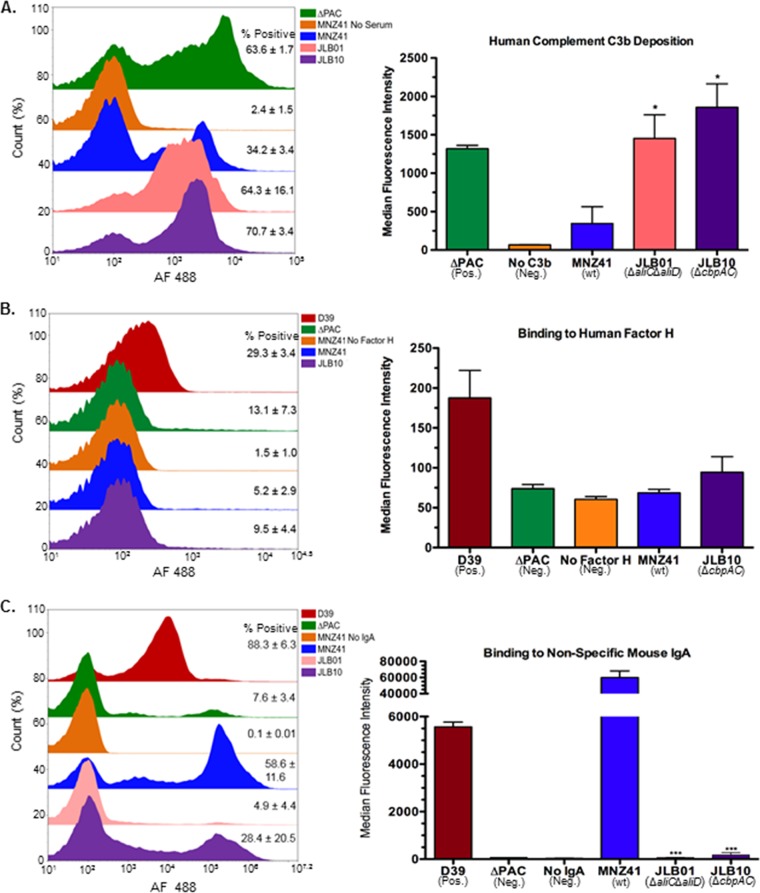
Flow cytometry analysis of host immune factors binding to NESp surfaces. (A and B) Pneumococci were incubated with pooled normal human serum followed by biotinylated goat anti-human C3b (A) or purified biotinylated human factor H (B). (C) Bacterial cells were incubated with nonspecific mouse IgA followed by biotinylated goat anti-mouse IgA. The cells were subsequently stained with streptavidin-conjugated Alexa Fluor 488 and subjected to flow cytometry. A total of 50,000 events were collected, and percent positive cells was calculated by gating fluorescence higher than background. The averages of percent positive cells ± standard error of the mean are listed to the right of representative histograms in overlays, and the mean fluorescence intensities are represented in bar graphs. D39 is a type 2 pneumococcal strain expressing PspC/CbpA, and ΔPAC is a D39 derivative strain with deletions of *ply*, *pspC*, and *pspA*. NESp CbpAC decreased human C3b deposition on NESp surface (A), but decreased deposition was not a result of binding to human factor H (B). NESp CbpAC variant significantly binds to nonspecific murine IgA (C). Data are representative of two independent experiments. Error bars denote standard errors of the means. Values that are significantly different are indicated by asterisks as follows: *, *P* < 0.05; ***, *P* < 0.001.

PspC of encapsulated pneumococci binds to host secretory immunoglobulin A (sIgA), as well as factor H that has been shown to decrease C3b deposition on the pneumococcal surface (30, 31). Sequence divergence predicts CbpAC binds to the Fc portion of IgA, rather than binding to the secretory component of sIgA (29). Furthermore, pneumococcal survival upon exposure to whole blood (Fig. 7A) suggests that NESp has developed a mechanism to resist early innate immune responders present in blood. To assess whether CbpAC provides functions similar to encapsulated PspC, we used flow cytometry to examine binding of host immune factors to the surfaces of viable pneumococci (Fig. 8). A deletion of *cbpAC* significantly increased (70.7% ± 3.4% positive) C3b deposition (Fig. 8A) (*P* < 0.05) compared to C3b deposited on the surface of strain MNZ41 (34.2% ± 3.4% positive). Strain JLB01 that expressed CbpAC at significantly lower levels than those of MNZ41 (Table 3) also had increased C3b deposited on its surface (64.3% ± 16.1% positive) (*P* < 0.05). However, factor H did not inhibit C3b deposition on NESp surfaces, as NESp was not able to bind to factor H (Fig. 8B). NESp CbpAC was also important for binding to nonspecific IgA (Fig. 8C), as a deletion of *cbpAC* resulted in decreased binding to nonspecific IgA (28.4% ± 20.5% positive) (*P* < 0.001) compared to MNZ41 (58.6% ± 11.6% positive).

## DISCUSSION

Our study demonstrates that AliC and AliD are essential for mucosal colonization and infection. Moreover, these oligopeptide binding proteins are required for persistence during invasive disease. As the pathogenic potential of NESp is poorly understood, this study elucidates AliC and AliD as mediators of NESp pathogenesis. Our *in vitro* analyses show that the presence of AliC and AliD results in a decrease of both adhesion to epithelial cells and *in vitro* biofilm formation, while increasing the expression of a critical cytolysin, Ply. These results correlate with a previous study that revealed that invasive pneumococcal strains produce more Ply and prefer a planktonic, less adhesive lifestyle (32). Also, the switch from an adhesive state to a more dynamic, virulent state has been previously shown in both Gram-positive bacteria and Gram-negative bacteria through quorum sensing coordinated with global gene regulation (33, 34). In the majority of our studies, a deletion of both *aliC* and *aliD* resulted in substantial phenotypic differences compared to a single deletion of *aliC* or *aliD*. Thus, it is likely that each oligopeptide binding protein serves separate functions, which exhibit a combined additive effect on pneumococcal physiology and sensing of external stimuli. Furthermore, these separate functions may be advantageous within different host compartments that provide a divergent stimulus. To our knowledge, *aliC* has been found in the *cps* locus only in the context of *aliD*, but *aliD* has been identified in the absence of *aliC* in the *cps* locus of encapsulated pneumococci and other *Streptococcus* genera, suggesting that AliD provides a more essential, conserved function (13, 35). Ply production (Fig. 3A) and NESp survival in whole blood (Fig. 6A) were greatly reduced when AliD was absent, thus signifying that AliD has a more dominant role in invasive disease.

Our comparative proteome data (Table 3; also see Data Set S1 in the supplemental material) indicate that oligopeptide import involving AliC and AliD serves as both a nutritional source and regulator of numerous virulence factors. Overall, the absence of AliC and AliD resulted in a starvation state in JLB01 cells. Proteins involved in nucleic acid synthesis, amino acid synthesis, protein synthesis, and protein folding were all downregulated in strain JLB01 compared to their levels in strain MNZ41. Furthermore, proteins involved in breaking down molecules for energy, protecting DNA during a starvation response, and inhibiting protein translation were upregulated in JLB01 cells. Interestingly, proteins associated with virulence in encapsulated bacteria, such as the PspC variant (CbpAC, NCBI accession no. EPD17685.1), NADH oxidase (EPD21629.1), and pyruvate oxidase (EPD21778.1), were upregulated in strain MNZ41 compared to strain JLB01 (31, 36–38). However, what occurs between substrate import and phenotype modification through protein dysregulation remains unknown. The imported oligopeptide substrates may alter regulation through direct recognition of DNA or through indirect signal transducing methods involving protein or RNA intermediates. Further studies are needed to identify direct interactions of oligopeptide substrates with intracellular machinery.

Even so, our *in vivo* data exposes the significance of AliC and AliD in both early colonization of a host and subsequent persistence. The presence of AliC and AliD improved murine nasopharyngeal colonization during initial colonization and as late as 5 days postinfection (Fig. 4A and B). AliC and AliD were also shown to be essential for persistence during an active lung infection (Fig. 4D). Likewise, chinchilla OM was significantly enhanced when AliC and AliD were present (Fig. 5A), with bacteremia occurring in chinchillas infected with NESp expressing AliC and AliD. Furthermore, virulence during OM was nearly abrogated when AliC and AliD or CbpAC alone was absent (Fig. 5C and 7D). Factors other than increased adherence to cells are likely aiding in colonization, as no differences in adherence to epithelial cells were observed in the MNZ85 background and increased adherence was observed when *aliC* and *aliD* were deleted from the MNZ41 background (Fig. 2). Also, CbpAC increased adherence to epithelial cells but had no significant effect on murine colonization (Fig. 7). Since NESp invades epithelial cells at a low rate (Fig. 2) and naturally lacks a protective capsule, NESp must be able to endure the extracellular association with pharyngeal and pulmonary cells. In a nutrient-deprived and complete system such as the environment in the murine nasopharynx and lungs, as well as in the chinchilla bulla, AliC and AliD are likely altering bacterial physiology in response to the external environment. Since there were no significant differences in growth between strains MNZ41 and JLB01 *in vitro* (Fig. S3), proteomic variances within the cells may be contributing to fitness within a host. Accordingly, increased biofilm formation in wt NESp during OM (Table 2) is probably a result of increased fitness leading to increased pneumococcal densities within the host (Fig. 5A and Fig. S2B). Additionally, Ply has been shown to be an important factor in the initial colonization of the murine nasopharynx (39). Although Ply was not implicated as significantly differentially expressed during stringent proteomic analysis (Table 3 and Data Set S1), small differences were amplified by ELISA detection (Fig. 3A and Fig. S1C) and were shown to impact cytolytic activity (Fig. 3B and Fig. S1D). Thus, increased Ply protein levels in strain MNZ41 in comparison to isogenic mutants may have influenced colonization of the murine nasopharynx, allowing for a higher recovery of pneumococci at 1 to 2 days postchallenge and enhanced persistence at 5 days postchallenge. Moreover, NADH oxidase that was upregulated in strain MNZ41 compared to that in the double mutant (Table 3) has also been shown to be important for murine colonization and invasive disease (37). NESp CbpAC was shown to be essential only during chinchilla OM (Fig. 7), which demonstrates that phenotypic effects of NESp virulence factors are conditional and dependent on specific niches in the host. Nonetheless, Ply, NADH oxidase, and PspC variants are virulence proteins shared by pathogenic encapsulated and nonencapsulated pneumococci. Accordingly, these proteins may serve as preventive and therapeutic options that target a broadened pneumococcal population.

The development of systemic disease during OM in chinchillas infected with wt NESp gives further evidence of the significance of AliC and AliD in NESp invasive disease. The isolation of NESp expressing AliC and AliD during IPD cases, as well as the observation of systemic disease in chinchillas infected by strains MNZ85 and MNZ41, directed us to investigate possible immune evasion mechanisms that could allow NESp to survive and even replicate in whole blood (Fig. 6A). As neutrophils are the dominant immune cell type in blood, we chose to examine NESp interactions with neutrophils on a defined surface. Also, complement serum proteins are important mediators of innate immunity that are deposited on the surfaces of invading bacteria (40). All pathways of complement activation converge on complement factor C3b, resulting in opsonophagocytosis and clearance of invasive bacteria (41). NESp possessing AliC and AliD were significantly more resistant to killing by neutrophils (Fig. 6B) and exhibited decreased C3b deposited on their surfaces (Fig. 8A) compared to isogenic mutants. Ply has been previously shown to lyse neutrophils (42), which may be enhancing the ability of strain MNZ41 to resist killing by neutrophils. However, other factors or biological processes are facilitating resistance to killing by PMNs, as a deletion of *aliC* did not significantly alter Ply production (Fig. 3) but resulted in decreased NESp survival when exposed to neutrophils. These factors or processes aiding in NESp survival may include bacterial proteins that induce apoptosis of neutrophils or help bacterial resistance to phagocytosis or subsequent killing. For inhibiting C3b deposition, it is probable that AliC and AliD are able to bind to host regulators of complement deposition or that the imported oligopeptide substrates alter expression of pneumococcal surface proteins that bind to or function as inhibitors of complement deposition. Surprisingly, sequenced NESp isolates expressing AliC and AliD do not encode PspC, also known as CbpA (43). PspC is a pneumococcal surface protein that binds host factor H, thus allowing inhibition of C3b deposition (41, 44, 45). Since the NESp PspC variant CbpAC was upregulated in strain MNZ41 compared to strain JLB01 (Table 3), we investigated whether CbpAC was functioning similar to encapsulated PspC by inhibiting C3b deposition through binding to factor H. We observed that CbpAC significantly reduced C3b deposition (Fig. 8A), but the decreased deposition was not a consequence of binding to full-length factor H (Fig. 8B). Thus, NESp CbpAC may be preventing C3b deposition by binding to truncated versions of factor H or through other mechanisms that may be specific to one of the complement pathways before they converge on C3b deposition. Remarkably, we were able to confirm that CbpAC does bind to nonspecific IgA (Fig. 8C), and binding of IgA may compensate for lack of capsule by creating an IgA shield that could prevent recognition or clearance by host immune cells.

Finally, currently licensed vaccines elicit limited protection against encapsulated pneumococci and do not prevent NESp infections. Thus, a protein-based vaccine, including proteins expressed by both encapsulated and nonencapsulated pneumococci would confer a broader protection against invasive populations of pneumococci. NESp bacteria with increased antibiotic resistance and mobile genetic elements are clustering and becoming highly prevalent (29, 43, 45). Current capsule-targeted vaccines selecting for outgrowth of NESp subpopulations will continue to potentiate the risk of invasive disease associated with NESp strains. Previous studies have shown that importer orthologs of AliC and AliD are immunogenic and serve as prominent vaccine candidates (46, 47). Hence, future studies of the immunogenicity of AliC and AliD both exclusively and in combined formulations with other virulence proteins of pneumococci are warranted. Also, PspC immunization has been shown to be protective against encapsulated pneumococcal disease (48). NESp strains that do not express AliC or AliD contain the gene *pspK* in the capsule locus, which encodes a pneumococcal surface protein that has high sequence identity to the R1 region of encapsulated PspC (13). A PspC variant was shown to be upregulated in a NESp strain expressing AliC and AliD (strain MNZ41) in comparison to the isogenic double mutant JLB01. Thus, a PspC fragment that shares similarity in encapsulated and NESp strains may also serve as a vaccine candidate that could elicit protection against a broader population of pneumococci. Altogether, as surveillance studies continue to reveal the rapid adaption of pneumococci and decreasing efficacy of polysaccharide-targeted vaccines, studies in alternative immunization methods will remain a necessity for prevention of pneumococcal disease.

## MATERIALS AND METHODS

### Bacterial growth.

All pneumococcal strains were incubated at 37°C with 5% CO_2_ and cultured on either sheep blood agar (BA) or in Todd-Hewitt medium with 0.5% yeast extract (THY) with respective antibiotic selection. The strains utilized in this study and descriptions of the strains are given in Table 1.

### DNA mutagenesis.

Isogenic mutants of MNZ85 and MNZ41, LEK08 and JLB01, respectively, were generated by allelic replacement of the capsule locus with a spectinomycin resistance cassette amplified from strain LEK05 as previously described (49). MNZ41 isogenic single mutations of *aliC* (JLB02) and *aliD* (JLB04) were created by allelic replacement of *aliC* or *aliD* with a kanamycin resistance cassette or spectinomycin resistance cassette, respectively. An isogenic deletion of the *pspC* variant (*cbpAC*) in strain MNZ41 (JLB10) was generated through allelic replacement of *cbpAC* with a spectinomycin resistance cassette.

### Biofilm viability and production.

A 24-well plate was seeded with 10^5^ CFU/ml of the nonencapsulated *Streptococcus pneumoniae* (NESp) strains in biofilm media (THY, 8 U/ml catalase, and 10% horse serum) and incubated overnight. To determine biofilm formation, the medium was removed from wells after overnight growth, and biofilms were stained with 350 µl of 0.1% crystal violet at room temperature for 30 min. Unbound crystal violet was carefully removed, and biofilm-associated crystal violet was solubilized by the addition of 1 ml of 100% ethanol to each well with shaking at room temperature for 10 min. Solubilized crystal violet was plated on a 96-well plate, and biofilm production was determined by reading the optical density at 630 nm (OD_630_). After removal of excess crystal violet, images of biofilm formation were captured at a ×10 magnification using a Nikon Eclipse 50i microscope with DS camera control unit DS-U2 (version 4.4) and NIS-Elements BR 3.0 software. To determine viability, medium was removed from wells after overnight growth, and biofilms were suspended in 500 µl of phosphate-buffered saline (PBS), serially diluted, and plated on BA to enumerate pneumococcal CFU.

### Adherence and invasion of human epithelial cells.

Human Detroit 562 pharyngeal (ATCC CCL-138, obtained from Justin Thornton, Mississippi State University, USA) and A549 pulmonary (ATCC CCL-185, obtained from Stephen Stray, University of Mississippi Medical Center, USA) epithelial cells were cultured to approximately 90% confluence (~3 × 10^5^ cells/ml for Detroit 562 cells and ~1.5 × 10^5^ cells/ml for A549 cells) in 10% fetal calf serum (FCS)-supplemented Eagle’s minimal essential medium (EMEM) with 100 µg/ml penicillin, 50 µg/ml streptomycin, and 100 µg/ml amphotericin B in 24-well plates at 37°C with 5% CO_2_. Human epithelial cells that had reached at least 90% confluence were washed three times with PBS. Approximately 10^7^ pneumococci were added to each well and allowed to adhere for 30 min or invade for 2 h at 37°C with 5% CO_2_. Following incubation, epithelial cells were washed three times with PBS. To assess pneumococcal adherence, human epithelial cells were lifted with 100 µl of 0.25% trypsin-EDTA, suspended in 900 µl of PBS, and plated on BA to enumerate adherent pneumococcal CFU. For invasion, epithelial cells were incubated with 1 ml of EMEM containing 100 µg/ml penicillin, 50 µg/ml streptomycin, and 100 µg/ml amphotericin B for an additional hour to eliminate extracellular bacteria. Epithelial cells were subsequently washed three times with PBS, lifted with 100 µl of 0.25% trypsin-EDTA, lysed with 100 µl of cold 0.0125% Triton X-100, and plated on BA to enumerate intracellular pneumococci. Recovered pneumococcal CFU were adjusted by standardizing inocula to 10^7^ CFU.

### Ply protein production and activity.

NESp strains were cultured to mid-log phase in THY, and 1 ml of bacterial culture was pelleted. Bacterial cell lysates were prepared by suspending cell pellets in 100-µl volumes of lysis buffer (0.01% sodium dodecyl sulfate, 0.1% sodium deoxycholate, and 0.015 M sodium citrate), incubating for 30 min at 37°C, and diluting in 100 µl of PBS. Prepared lysates were standardized to equivalent total protein concentrations by determining total protein concentration in cell lysates through standard bicinchoninic acid (BCA) assay protocol (Sigma-Aldrich) and diluting with PBS to equivalence. To measure intracellular Ply levels, prepared lysates were used to coat a 96-well plate in triplicate overnight, and the coated plate was used in an indirect enzyme-linked immunosorbent assay (ELISA) with primary rabbit anti-Ply serum (provided by Mary Marquart, University of Mississippi Medical Center, USA). Donkey anti-rabbit IgG (GE Healthcare Life Sciences) conjugated to alkaline phosphatase was used as the secondary antibody. The 96-well plate was read at OD_405_ on an xMark spectrophotometer to determine Ply concentration relative to the concentration in strain MNZ41. To measure Ply activity, standardized lysates were plated in triplicate in a 96-well round bottom plate and 50 µl of 2% sheep red blood cells (RBCs) were added to each well. PBS served as a negative control for RBC lysis, and 1% Triton X-100 served as a positive control for RBC lysis. Plated blood mixtures were incubated for 30 min at 37°C, and nonlysed RBCs were subsequently collected by centrifugation at 500 × *g* for 5 min. To assay for lysed RBCs, 100 µl of supernatant was transferred to a 96-well flat bottom plate and spectrophotometrically measured at OD_450_.

### Murine nasopharyngeal colonization.

Six- to eight-week-old C57BL/6 mice were anesthetized with isoflurane and intranasally challenged with 10 µl of PBS containing 10^7^ CFU of pneumococci. For cocolonization studies, mice were challenged with 10 µl of a 1:1 mixture containing 10^7^ CFU of pneumococci (5 × 10^6^ CFU of EF3030 and 5 × 10^6^ CFU of strain MNZ41). At 1, 2, and 5 days after challenge, the mice were euthanized, the murine nasopharynx was washed with 200 µl of PBS, and nasopharyngeal tissue was collected. Bacterial load was enumerated by plating nasal washes and tissue homogenates on BA with 5 µg/ml gentamicin to estimate log CFU/mouse. For cocolonization studies, nasal washes and tissue homogenates were also plated on BA containing 50 µg/ml trimethoprim to enumerate recovered MNZ41 CFU. Recovered EF3030 CFU was calculated by subtracting MNZ41 counts from total recovered bacterial CFU enumerated on BA containing 5 µg/ml gentamicin.

### Murine pulmonary infections.

Adult C57BL/6 mice (weight, 20 to 25 g) were anesthetized with 500 µl Avertin (6.25 mg tribromoethanol dissolved in 2.5% amylene hydrate solution) and intratracheally inoculated with 50 µl of PBS containing 10^7^ CFU of pneumococci. The mice were euthanized, and the lungs were collected 2 days postinfection. The lungs were homogenized in 2 ml PBS and plated on BA to enumerate recovered log CFU/mouse.

### Chinchilla acute otitis media.

Pneumococcal virulence during otitis media (OM) was assessed as previously described (49). Briefly, young adult chinchillas (*Chinchilla lanigera*) (body weight, 400 to 500 g) were inoculated via transbullar injection with approximately 10^7^ CFU of pneumococci. Chinchillas were euthanized 4 days postinfection, and tympanic pathology was assessed through otoscopic examination and biofilm formation within each bulla. Middle ear effusion was collected, each bulla was lavaged with 1 ml PBS, and each individual bulla was homogenized in 10 ml of PBS. All CFU were enumerated on BA with 5 μg/ml gentamicin. Combined effusion, lavage, and homogenate counts were used to estimate the log CFU/bulla.

Tympanic pathology was scored through otoscopic examination both before and after infection, as well as biofilm formation after euthanasia. Otoscopic examination of the tympanic membrane was scored as follows: 0 for no inflammation, 1 for inflammation, 2 for effusion, and 3 for tympanic rupture. Biofilm formation was scored as follows: 0 for no visible biofilm formation, 1 for biofilm formation on the surface of the bulla, 2 for the biofilm traverses the bulla, and 3 for the biofilm traverses the bulla with thickening. Otoscopic and biofilm scores are listed in Table 2.

### Chinchilla whole blood killing.

Whole blood was collected from the saphenous veins of healthy adult chinchillas. Approximately 10^5^ CFU pneumococci were incubated in 150 µl of fresh, heparinized chinchilla whole blood, and the blood mixture was rotated end over end for 3 h at 37°C. Initial inocula and blood mixtures following 3-h incubation were serially diluted and plated on BA with 5 μg/ml gentamicin. Percent survival was calculated by dividing the CFU enumerated after 3-h incubation by the respective inoculum (CFU) and multiplying by 100.

### Human neutrophil (PMN) surface killing.

To isolate human polymorphonuclear leukocytes (PMNs), 60 ml of freshly collected human whole blood was gently mixed with 12 ml of Lymphoprep (Stemcell Technologies) and allowed to settle for 30 min. The lymphocyte-enriched supernatant was centrifuged at 225 × *g* for 10 min at 25°C to collect lymphocytes. Pelleted cells were suspended in 6 ml of PBS, and the cell suspension was overlaid on 3 ml of Lymphoprep, followed by centrifugation at 300 × *g* for 40 min at 25°C. Hypotonic lysis to remove residual RBCs was performed by suspending cell pellets in 7-ml volumes of ice-cold 0.2% NaCl, incubating for 25 s on ice, followed by the addition of 7 ml of 1.6% NaCl to restore tonicity. Neutrophils were collected by centrifugation at 225 × *g* for 10 min at 4°C, and cell pellets were suspended in 3 ml of ice-cold Hanks balanced salt solution (HBSS). Neutrophil concentration was enumerated using a hemocytometer and adjusted by dilution in HBSS.

Pneumococcal survival upon exposure to human neutrophils was performed as previously described (50) with modifications. Briefly, 10 µl of 5 × 10^3^ CFU/ml pneumococci was spotted with six replicates on BA. After the spotted bacterial cells had dried, 20 µl of 2 × 10^6^ cells/ml of freshly isolated neutrophils (multiplicity of infection [MOI] of 800) was overlaid on each bacterial replicate. A duplicate plate spotted with pneumococci was overlaid with 20 µl HBSS and served as the control. The plates were incubated for 24 h, and pneumococcal CFU was enumerated to determine percent pneumococcal survival. Percent survival was calculated by dividing the PMN-treated plate CFU by the control plate CFU and multiplying by 100.

### Comparative proteomic analysis.

Four replicates each of pneumococcal strains MNZ41 and JLB01 were subjected to proteomic analysis. The cultures were grown to mid-log phase (OD_600_ of 0.25), pelleted, and lysed with 100 µl hexafluoroisopropanol (HFIP). HFIP was removed using vacuum centrifugation, and dried lysates were rehydrated with the addition of 10 µl of 100 mM dithiothreitol (DTT) in 100 mM ammonium bicarbonate (ABC) and placed at 85°C for 5 min to reduce disulfide bonds. Samples were alkylated by the addition of 10 µl iodoacetamide in 100 mM ABC and placed at room temperature in the dark for 30 min. Two micrograms of trypsin in 200 µl of 100 mM ABC was added to each sample and placed at 37°C overnight for enzymatic digestion. After trypsin digestion, samples were desalted using a peptide reverse-phase microtrap (Michrom BioResources), dried, and suspended in 20 µl of 2% (vol/vol) acetonitrile and 0.1% (vol/vol) formic acid. A Dionex UltiMate 3000 (Thermo Scientific) high-performance liquid chromatography (HPLC) system was configured for reverse-phase chromatography with a C_18_ column packed with Halo 2.7-µm, 90-Å C_18_ material (MAC-MOD Analytical) and a flow rate of 333 nl/min. Separation of peptides was performed using a gradient from 2 to 50% acetonitrile over 4 h, followed by a 15-min wash with 95% acetonitrile, and a 15-min equilibration with 2% acetonitrile. The eluate from HPLC was analyzed by an LTQ Velos Pro mass spectrometer (Thermo Scientific) using nanospray ionization and parameters of one mass spectrometry (MS) scan followed by 10 tandem MS (MS/MS) scans of the five most intense peaks. MS/MS scans were performed in pairs: a collision-induced dissociation (CID) fragmentation scan followed by a higher-collision dissociation (HCD) fragmentation scan of the same precursor *m/z*. Dynamic exclusion was enabled with a mass exclusion time of 3 min and a repeat count of 1 within 30 s of initial *m/z* measurement. Spectra were collected over the entirety of each 4-h chromatography run. Raw mass spectra were converted to MGF format using MSConvert, part of the ProteoWizard software library (51). X!tandem 2013.09.01.1 (52) and OMSSA (open mass spectrometry search algorithm) (53) algorithms were employed to perform MS/MS spectrum matching. Precursor and fragment mass tolerance were set to 0.25 Da for both the OMSSA and X!tandem algorithms. Trypsin cleavage rules were used for both algorithms with up to two missed cleavages. Amino acid modifications searched consisted of single and double oxidation of methionine, oxidation of proline, N-terminal acetylation, carbamidomethylation of cysteine, deamidation of asparagine and glutamine, and phosphorylation of serine, threonine, and tyrosine. X!tandem xml and OMSSA xml results were filtered using Perl to remove any peptide matches with an E value of >0.01 as well as proteins identified by a single peptide sequence. The protein fasta database for strain MNZ41 (GenBank accession no. ASJQ00000000.1) was downloaded from the National Center for Biotechnology Information reference sequences (NCBI RefSeq), and a randomized version of the MNZ41 fasta was concatenated to the original as a way to assess data set quality and calculate false-discovery rates. The mass spectrometry proteomic data have been deposited to the ProteomeXchange Consortium via the PRIDE partner repository (54) with the data set identifier PXD005178.

Strain MNZ41 and JLB01 proteomes were evaluated using differential expression analysis based on peptide precursor mass ion current performed using the Perl programing language as described previously (55). Briefly, for each identified peptide, elution curves were integrated and organized by protein and then technical replicate. Permutation tests were performed to determine whether any differences between strains MNZ41 and JLB01 were significant. A *P* value of ≤0.05 was used to indicate significance.

### Flow cytometry.

Pneumococcal cells were grown to mid-log phase, and 10^7^ CFU of pneumococci were pelleted by centrifugation and washed twice with 1 ml of PBS for all experiments. For human C3b deposition, bacterial cells were suspended in 10% pooled human serum in gelatin-based veronal buffer (0.15 mM CaCl_2_, 141 mM NaCl, 0.5 mM MgCl_2_, 0.1% gelatin, 1.8 mM sodium barbital, and 3.1 mM barbituric acid [pH 7.3 to 7.4]) and allowed to incubate with rotation for 30 min at 37°C. Pneumococci were subsequently collected, washed twice with PBS, and incubated with 200 µl of 1 μg/ml biotinylated anti-human C3b (Cedarlane Laboratories Limited) for 30 min on ice. A serotype 2 *ply*, *pspA*, and *pspC* deletion mutant (ΔPAC) known to strongly bind complement C3b was used as the positive control (31). MNZ41 cells incubated without serum were used as the negative control. For binding to factor H, bacterial cells were suspended in 200 µl of 20 µg/ml biotinylated factor H (obtained from Michael K. Pangburn, University of Texas Health Science Center, USA) for 1 h on ice. Strain D39 (type 2) expressing PspC known to bind to factor H (56) was used as a positive control for binding, and MNZ41 cells not incubated with factor H were used as a negative control. For binding to nonspecific mouse IgA, bacterial cells were incubated with 200 µl of 10 µg/ml nonspecific mouse IgA (Southern Biotech) for 1 h on ice, washed twice with 500 µl PBS, followed by 200 µl of 5 µg/ml biotinylated goat anti-mouse IgA for 30 min on ice. MNZ41 cells incubated without mouse IgA were used as a negative control. For flow cytometry analysis, bacteria were collected, washed with PBS, stained with streptavidin-conjugated Alexa Fluor 488 (Invitrogen), and incubated on ice for 30 min with rotation in the dark. Following incubation, cells were collected, washed with PBS three times, suspended in 500 µl PBS, and analyzed by a NovoCyte flow cytometer with NovoSampler.

### Ethics statement.

All animal studies were performed in accordance with protocols reviewed and approved by the University of Mississippi Medical Center Institutional Care and Use Committee (protocol 1361). The institution has an approved Animal Welfare Assurance on file with the Division of Assurances, Office of Laboratory Animal Welfare (OLAW) in compliance with the Public Health Service (PHS) Policy on Humane Care and Use of Laboratory Animals.

### Statistics.

Results were analyzed using the InStat program (GraphPad PRISM 4 Software, San Diego, CA). When two different strains were being compared, the nonparametric Mann-Whitney *t* test was used for analysis. One-way analysis of variance (ANOVA) with Bonferroni posttest was applied when comparing three or more strains. Flow cytometry statistics were provided by NovoExpress software (ACEA Biosciences, San Diego, CA). Proteome statistics were performed as described above in “Comparative proteomic analysis.”
