# Supplementary material for: Mucosal Infections and Invasive Potential of Nonencapsulated Streptococcus pneumoniae Are Enhanced by Oligopeptide Binding Proteins AliC and AliD
Source: mBio. 2018 Jan 16;9(1):e02097-17. doi: 10.1128/mBio.02097-17 (PMC5770551; doi:10.1128/mBio.02097-17)
Supplement: FIG S1 [file mbo001183686sf1.pdf]

FIG S1

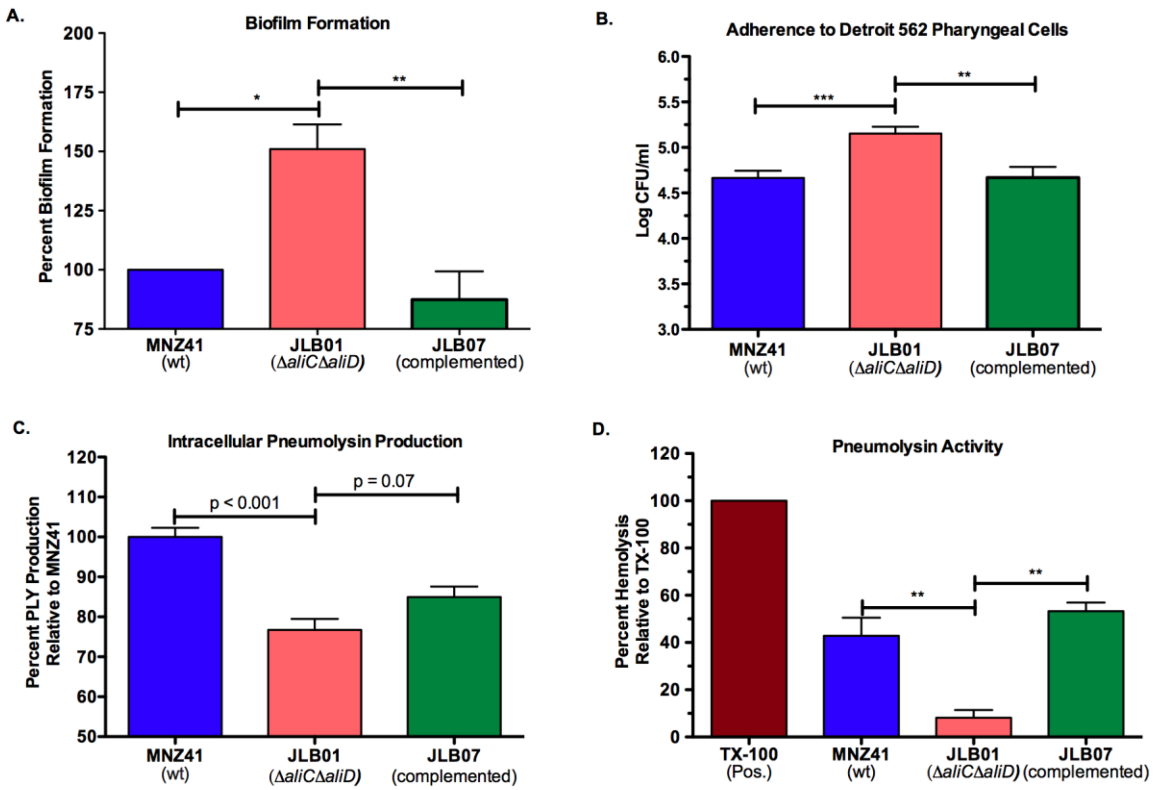

FIG S1 Genetic complementation restores in vitro phenotypes comparable to NESp wt strain MNZ41. MNZ41 isogenic mutant encoding *aliC* but not *aliD* was transformed with pABG5::*aliD* to produce JLB07 for complementation studies. Biofilm formation (A) and adherence to human epithelial cells (B) were significantly decreased in JLB07 similar to wt levels. Hemolytic activity (D) significantly increased in JLB07 comparable to wt, with a strong trend toward increased Ply production also observed (C). Data represent two independent studies performed in triplicate. Error bars represent standard error of the mean. (* = p<0.05, ** = p<0.01, ***p<0.001)
