# Supplementary material for: Mucosal Infections and Invasive Potential of Nonencapsulated Streptococcus pneumoniae Are Enhanced by Oligopeptide Binding Proteins AliC and AliD
Source: mBio. 2018 Jan 16;9(1):e02097-17. doi: 10.1128/mBio.02097-17 (PMC5770551; doi:10.1128/mBio.02097-17)
Supplement: FIG S2 [file mbo001183686sf2.pdf]

FIG S2

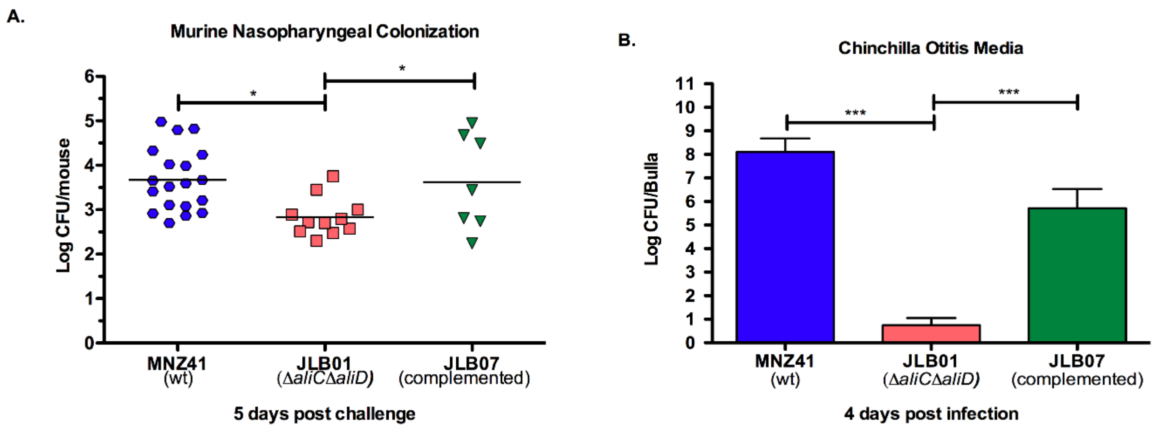

FIG S2 In vivo murine nasopharyngeal colonization and chinchilla otitis media phenotypes are restored after genetic complementation. Recovered JLB07 CFU during murine colonization at 5 days post challenge (A) and chinchilla otitis media at 4 days post infection (B) were significantly increased similar to wt levels. Data represent at least two independent studies. Error bars represent standard error of the mean. (* = $p<0.05$, *** = $p<0.001$)
