# Supplementary material for: Mucosal Infections and Invasive Potential of Nonencapsulated Streptococcus pneumoniae Are Enhanced by Oligopeptide Binding Proteins AliC and AliD
Source: mBio. 2018 Jan 16;9(1):e02097-17. doi: 10.1128/mBio.02097-17 (PMC5770551; doi:10.1128/mBio.02097-17)
Supplement: FIG S3 [file mbo001183686sf3.pdf]

FIG S3

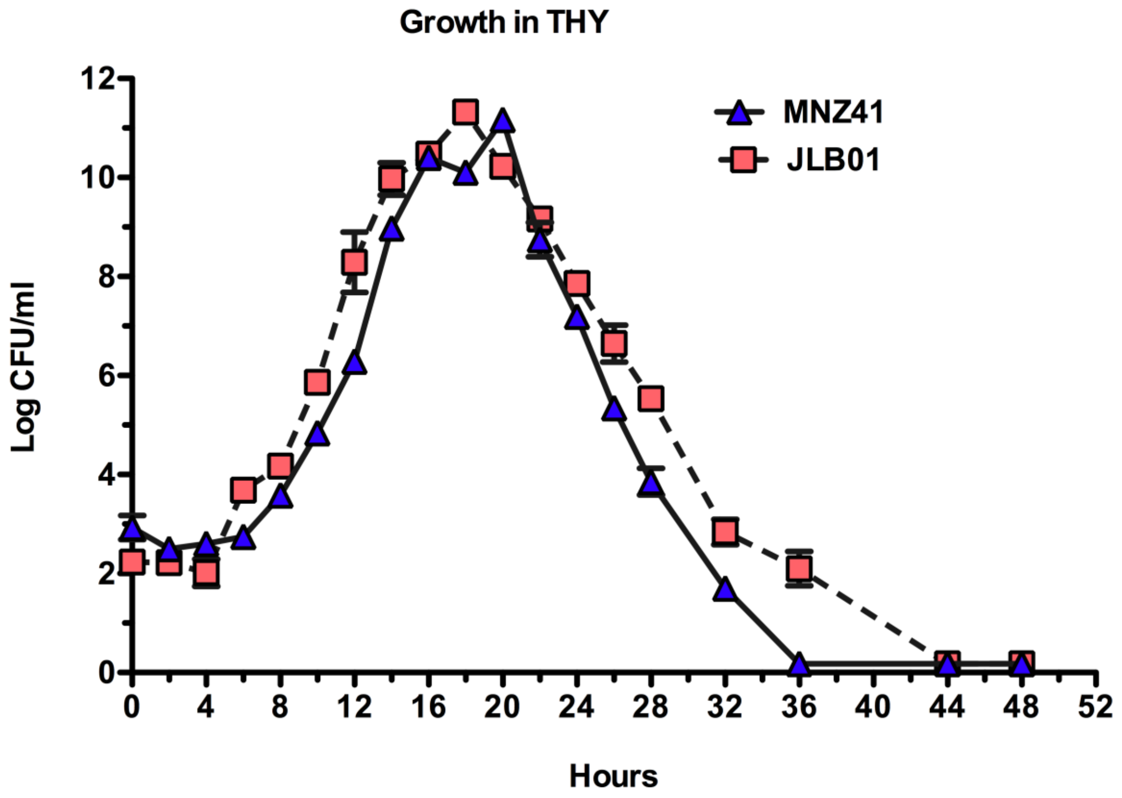

FIG S3 Growth curves of MNZ41 and JLB01 cultured in THY broth. Pneumococci were grown to mid-log phase and diluted to 100 CFU/ml in 10 ml THY for growth curve analysis. At indicated time points, 60 µl of culture was removed, diluted, and plated in duplicate to enumerate CFU/ml. There was no significant difference in growth between MNZ41 and JLB01. Data represent three independent studies. Errors bars represent standard deviation of the mean.
